# The role and mechanism of endothelial cell-specific molecule-1 in respiratory diseases

**DOI:** 10.3389/fphys.2026.1914718

**Published:** 2026-10-14

**Authors:** Ling Huang, Xiaofei Liu, Qianbiao Rao, Yingchun Zou, Yiqiong Wen, Talhah Arif, Yuanyuan Zheng, Shibo Sun

**Affiliations:** Department of Pulmonary and Critical Care Medicine, First Affiliated Hospital, Kunming Medical University, Kunming, China

**Keywords:** asthma, chronic obstructive pulmonary disease (COPD), endocan, inflammation, metabolic reprogramming

## Abstract

Endothelial cell-specific molecule-1 (ESM1) is a soluble dermatan sulfate proteoglycan and is mainly expressed in pulmonary endothelial cells. It is reported that ESM1 plays an important role in promoting cell proliferation, such as metabolic reprogramming, and inhibiting autophagy. In addition, ESM1 exerts a dual regulatory effect on inflammation. However, the mechanism of the dual regulatory effect of ESM1 on inflammation remains unclear. Moreover, evidence suggests that ESM1 is involved in multiple signaling pathways, such as the EGFR/ESM1, EGFR/HER3-Akt/ANGPT2, HGF/c-MET, VEGF-A/ESM1, mTOR, and ZEB1/PI3K/AKT pathways. Increasing studies suggested that ESM1 is involved in various respiratory diseases, such as lung cancer, obstructive sleep apnea hypopnea syndrome (OSAHS), pulmonary arterial hypertension (PAH), and acute lung injury (ALI)/acute respiratory distress syndrome (ARDS). In lung cancer, ESM1 promotes cancer cell proliferation through the EGFR, NF-κB, and HGF/c-MET signaling pathways. In OSAHS, ESM1 enhances inflammatory factor secretion and monocyte-endothelial cell adhesion through the HIF-1α/VEGF signaling pathway. In PAH, ESM1 may promote vascular remodeling and inflammation via a positive feedback loop with TNF-α. Conversely, in ALI/ARDS, ESM1 may inhibit leukocyte recruitment to reduce inflammation. However, the specific roles and mechanisms of ESM1 in chronic obstructive pulmonary disease (COPD), asthma, and pneumonia need to be further explored. Accordingly, ESM1 may serve as a potential therapeutic target for various respiratory diseases.

## 1 Introduction

Respiratory diseases are a leading cause of global morbidity and mortality, imposing a substantial burden on healthcare systems (Cao et al., 2025). Evidence suggests that pulmonary endothelial cells play an important role in respiratory diseases (Fließer et al., 2024). Pulmonary endothelial cells mediate lung homeostasis and pathological responses by secreting various bioactive factors, including cytokines, chemokines, and soluble proteoglycans (Vassiliou et al., 2020; Osorio-Valencia and Zhou, 2024). Therefore, targeting specific bioactive factors represents a promising therapeutic strategy for respiratory diseases (Li et al., 2022).

Endothelial cell-specific molecule-1 (ESM1), also known as endocan, is a soluble dermatan sulfate proteoglycan discovered from a human umbilical vein endothelial cells (HUVECs) cDNA library (Lassalle et al., 1996). Additionally, ESM1 is mainly expressed in the endothelial cells of pulmonary arterioles, venules, and alveolar capillaries, as well as in the intestine, heart, and placenta (Chew et al., 2019; Bovay et al., 2025; Chen et al., 2026). Evidence suggests that ESM1 not only promotes cancer cell proliferation and inhibits apoptosis, but also plays a crucial role in mediating inflammation (Gaudet et al., 2019; Zhang et al., 2021; Lu et al., 2022). Increasing studies suggest that ESM1 is closely related to respiratory diseases. It is reported that ESM1 promotes cancer cell proliferation and metastasis in lung cancer (Aliquò et al., 2023). In addition, ESM1 may promote inflammation in obstructive sleep apnea hypopnea syndrome (OSAHS), whereas ESM1 may inhibit inflammation in acute lung injury (ALI)/acute respiratory distress syndrome (ARDS) (Sun et al., 2019; Hureau et al., 2023). Accordingly, ESM1 may be a potential therapeutic target in respiratory diseases. This article reviewed the role and mechanism of ESM1 in respiratory diseases.

## 2 The structure of ESM1

The *ESM1* gene is located on human chromosome 5q11.2 and spans approximately 12 kb, consisting of 3 exons and 2 introns (Liu et al., 2024). ESM1 mRNA encodes a signal peptide composed of 19 amino acids and a mature polypeptide composed of 165 amino acids (De Freitas Caires et al., 2018). The signal peptide is recognized and bound by the cytosolic signal recognition particle (SRP), resulting in the signal peptide being cleaved by a signal peptidase within the endoplasmic reticulum, thereby producing a mature core polypeptide (Zhang et al., 2021, 2025). Subsequently, the core polypeptide chain is modified by O-glycosylation, attaching a linear dermatan sulfate (DS) glycosaminoglycan (GAG) chain to serine at position 137 (Ser137) (Zhou et al., 2024).

Mature ESM1 is a cysteine-rich molecule composed of a core polypeptide chain and a DS chain (**Figure 1**). In addition, ESM1 has an approximate molecular weight of 50 kDa (Liu et al., 2024). The core polypeptide chain contains an N-terminal cysteine-rich domain of 110 amino acids and a C-terminal phenylalanine-rich region. The N-terminal region contains an epidermal growth factor (EGF)-like domain (Pan et al., 2022). The cysteine rich domain is involved in blocking leukocyte adhesion and activation (Sarrazin et al., 2006). The phenylalanine rich region contributes to promoting cancer cell proliferation and invasion (Zhang et al., 2021). However, the function of the EGF like domain remains unclear. The DS chain plays roles in embryonic development, tumor progression, and other biological processes via binding to pro-angiogenic factors (Delehedde et al., 2013; Zhang et al., 2021).

**Figure 1:**
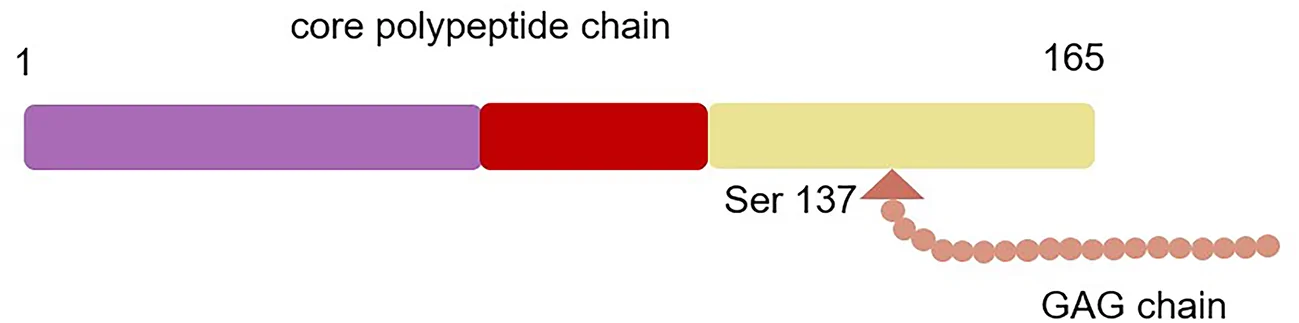
The protein structure of ESM1.

## 3 The biological function of ESM1

ESM1 exhibits diverse biological functions. Studies suggest that ESM1 promotes cell proliferation, metabolic reprogramming, endothelial-to-mesenchymal transition (EndMT), epithelial-mesenchymal transition (EMT), and oxidative stress. Additionally, ESM1 plays a crucial role in inhibiting autophagy and apoptosis. Furthermore, ESM1 exerts a dual regulatory effect on inflammation.

### 3.1 ESM1 and cell proliferation

It is indicated that ESM1 facilitates the proliferation of cancer cells. ESM1 promotes the expression of cyclin D1 and cyclin-dependent kinase 4 (CDK4), thereby accelerating the G1/S phase transition of the cell cycle to enhance the proliferation of colorectal cancer cells (Kang et al., 2012). Furthermore, ESM1 may promote lipid metabolism reprogramming to stimulate the proliferation of ovarian cancer cells (Li et al., 2024).

In addition, studies suggest that ESM1 may promote the proliferation of lymphatic endothelial cells and vascular smooth muscle cells (VSMCs) to drive cancer progression (Pan et al., 2022).

### 3.2 ESM1 and metabolic reprogramming

Metabolic reprogramming is a specific metabolic pattern in cancer cells (Zhou et al., 2025). Growing evidence demonstrates that ESM1 is involved in metabolic reprogramming. On the one hand, ESM1 drives lipid synthesis. Evidence indicates that ESM1 stimulates fatty acid synthesis in the hypoxic microenvironment (Zhang et al., 2024). In addition, ESM1 induces the expression of fatty acid synthase (FASN) and stearoyl-CoA desaturase 1 (SCD1), thereby promoting fatty acid synthesis in cancer cells and stimulating lipid synthesis in endothelial cells (Feng et al., 2024). Moreover, ESM1 induces a metabolic shift towards aerobic glycolysis to promote lipid synthesis. It is suggested that ESM1 promotes the SUMOylation of pyruvate kinase M2 (PKM2) in the hypoxic microenvironment, thereby triggering the Warburg effect to induce lactate and fatty acid synthesis in ovarian cancer (Zhang et al., 2024). On the other hand, ESM1 attenuates lipolysis. Studies suggest ESM1 may inactivate lipoprotein lipase (LPL) to inhibit lipolysis, thereby promoting abnormal intracellular lipid accumulation and driving lipid metabolic reprogramming in ovarian cancer (Li et al., 2024).

### 3.3 ESM1, autophagy, and apoptosis

Evidence suggests that ESM1 is involved in programmed cell death. ESM1 may reduce the expression of autophagy-related proteins to inhibit autophagy in liver cancer cells (Yang et al., 2017; Wang et al., 2025). In addition, ESM1 may block apoptotic signaling pathways to suppress cancer cell apoptosis (Yang et al., 2017; Huang et al., 2021).

### 3.4 ESM1, EndMT, and EMT

It is reported that ESM1 may promote EndMT, leading to aortic wall remodeling (Carrillo et al., 2011). Additionally, ESM1 may induce EndMT to promote renal fibrosis progression (Hung et al., 2020). Furthermore, ESM1 may induce EMT to promote the progression of cancer (Gu et al., 2021; Qi et al., 2024; Yang et al., 2024).

### 3.5 ESM1 and inflammation

Evidence suggests that ESM1 may promote inflammation. It is reported that ESM1 stimulates the secretion of interleukin-8 (IL-8) and monocyte chemoattractant protein (MCP-1) in endothelial cells, thereby facilitating leukocyte migration to exacerbate inflammation (Lee et al., 2014). In addition, ESM1 decreases tight junction proteins (occludin and zonula occludens-1) and induces endothelial cytoskeletal rearrangement, thereby increasing vascular endothelial permeability to promote inflammation (Lee et al., 2014). Moreover, ESM1 may enhance adhesion between monocytes and endothelial cells under chronic intermittent hypoxia (CIH) to promote inflammation (Sun et al., 2019). Additionally, some studies suggest that ESM1 activates the VEGF-A pathway by binding to fibronectin, promoting leukocyte transendothelial migration and increasing vascular permeability, thereby exacerbating inflammation (Rocha et al., 2014). However, some authors suggested that ESM1 suppresses inflammation. It is indicated that ESM1 inhibits lymphocyte function- associated antigen-1 (LFA-1) binding to intercellular adhesion molecule-1 (ICAM-1), thereby inhibiting leukocyte migration and reducing neutrophil infiltration to suppress inflammation (Béchard et al., 2001b; Gaudet et al., 2019). Moreover, ESM1 may reduce the secretion of TNF-α, IFN-γ, and IL-1β to attenuate inflammation (Zhang et al., 2018).

The dual roles of ESM1 in inflammation may be caused by multiple factors. Firstly, the pro-inflammatory and anti-inflammatory roles of ESM1 may be caused by different mechanisms of ESM1. ESM1 promotes endothelial cells to secrete inflammatory factors and adhesion molecules, thereby producing a pro-inflammatory effect (Lee et al., 2014), whereas ESM1 may inhibit the leukocyte migration and reduce neutrophil infiltration, thereby exerting an anti-inflammatory effect (Béchard et al., 2001b; Gaudet et al., 2019). Secondly, the dual roles of ESM1 in inflammation may result from the structure of ESM1. It is suggested that non-glycanated ESM1 may have a pro-inflammatory effect, while glycanated ESM1 has an anti-inflammatory effect (Yassine et al., 2015). In addition, cathepsin G secreted by activated neutrophils may cleave ESM1 (Yang et al., 2018), which may change the function of ESM1 in inflammation. Thirdly, the dual roles of ESM1 in inflammation may be caused by the difference of diseases. Studies suggest that ESM1 plays a pro-inflammatory role in sepsis, whereas ESM1 exerts an anti-inflammatory role in ALI (Lee et al., 2014; Gaudet et al., 2019).

### 3.6 ESM1 and oxidative stress

Evidence suggests that ESM1 promotes the generation of aberrant levels of NO in endothelial cells, thereby exacerbating oxidative stress (Kumar and Mani, 2021).

## 4 Signaling pathways of ESM1

It is suggested that ESM1 exerts biological effects through several signaling pathways, such as the EGFR/ESM1, EGFR/HER3-Akt/ANGPT2, HGF/c-MET, VEGF-A/ESM1, mTOR, and ZEB1/PI3K/AKT pathways (**Figure 2**).

**Figure 2:**
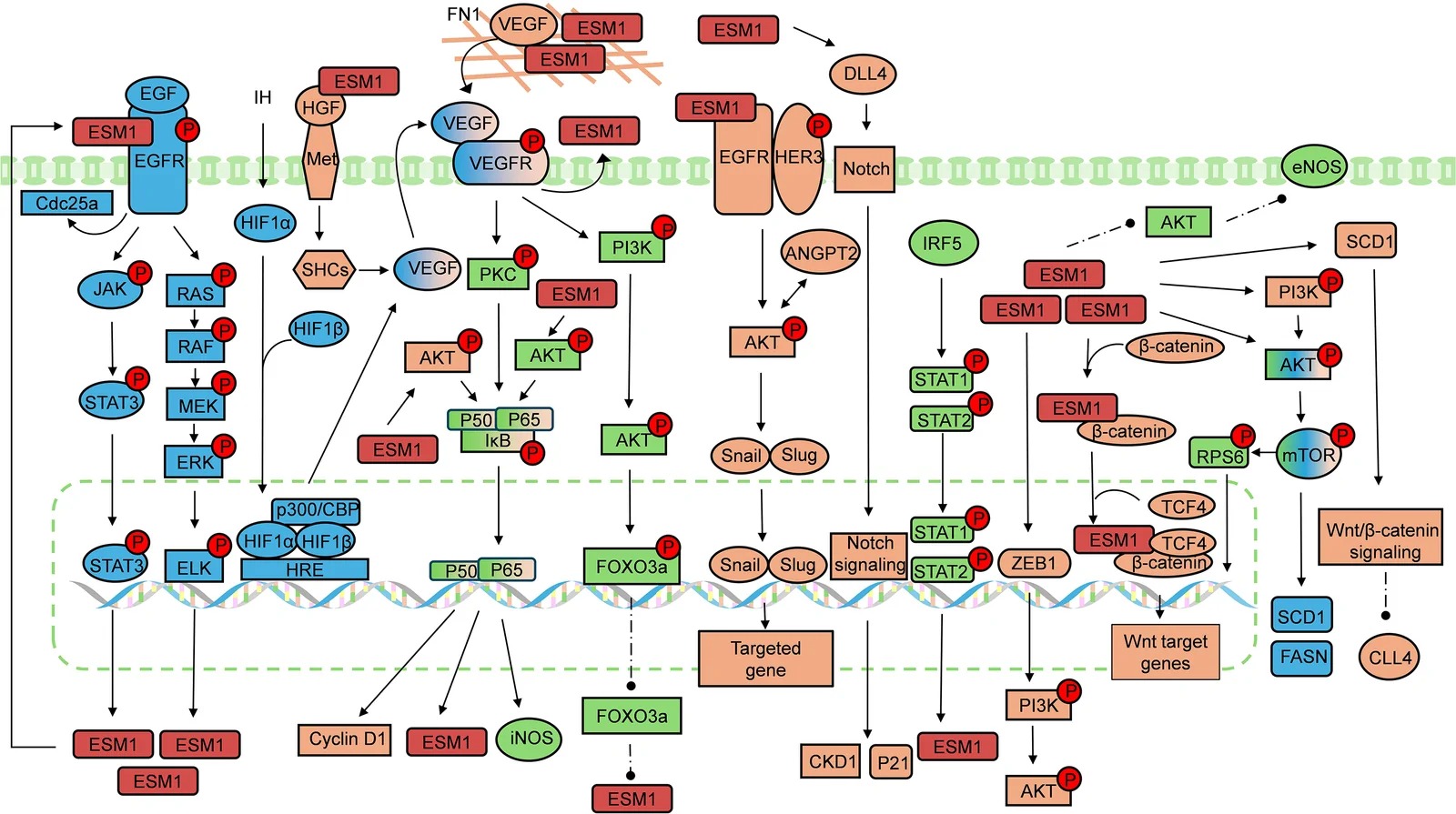
The signaling pathways related to ESM1. ESM1 involves several signaling pathways, including EGFR/ESM1, EGFR/HER3-Akt/ANGPT2, HGF/c-MET, VEGF-A/ESM1 signaling pathways, and so on. Blue: the signaling pathways identified in respiratory diseases (including lung cancer). Orange: the signaling pathways identified in non-respiratory cancers. Green: the signaling pathways identified in non-pulmonary contexts.

### 4.1 EGFR/ESM1 signaling pathway

EGFR is a tyrosine kinase receptor that plays a crucial role in the development and homeostasis of epithelial and epidermal tissues (Tydings et al., 2024). It is suggested that EGFR may phosphorylate JAK. Phosphorylated JAK subsequently phosphorylates STAT3, thereby stimulating ESM1 expression. Meanwhile, EGFR may phosphorylate MEK to phosphorylate ERK, thereby leading to the phosphorylation of ELK, ultimately EGFR promotes ESM1 expression. In addition, ESM1 may bind to the extracellular domain of EGFR to promote the binding of EGF to EGFR, thereby inducing the dissociation of Cdc25A from EGFR to activate the EGF/EGFR signaling pathway, ultimately promoting the proliferation of cancer cells (Yang et al., 2020).

### 4.2 EGFR/HER3-Akt/ANGPT2 signaling pathway

The EGFR/HER3-Akt/ANGPT2 signaling pathway plays a crucial role in promoting cancer progression (Yang et al., 2024). It is reported that ESM1 activates the EGFR/HER3-Akt/ANGPT2 signaling pathway to promote metastasis in cancer. ESM1 facilitates the formation of EGFR/HER3 heterodimers, thereby inducing HER3 phosphorylation to activate AKT. Activated AKT acts synergistically with ANGPT2 to stimulate the expression of transcription factors (such as Snail and Slug) to promote EMT in gastric cancer (Yang et al., 2024).

### 4.3 HGF/c-MET signaling pathway

The HGF/c-MET pathway regulates cell proliferation, migration, survival, morphogenesis, and differentiation (Yao et al., 2024). It is indicated that HGF binds to c-MET to recruit and activate Src homology 2 (SH2) domain-containing adaptor proteins (SHCs), thereby inducing VEGF-A expression to upregulate ESM1 expression (Li et al., 2019; Xia et al., 2025). Meanwhile, ESM1 activates the HGF/c-MET pathway to stimulate angiogenesis in cancer, ultimately forming a positive feedback loop between HGF and ESM1 (Yang et al., 2023a).

### 4.4 VEGF-A/ESM1 signaling pathway

VEGF-A/ESM1 signaling pathway plays an important role in angiogenesis (Li et al., 2023; Rachmadi et al., 2024). Evidence suggests that VEGF-A binds to vascular endothelial growth factor receptor-2 (VEGFR-2) to promote the phosphorylation of VEGFR-2, thereby inducing ESM1 expression (Zhou et al., 2024). Meanwhile, ESM1 binds to fibronectin 1 (FN1) in the extracellular matrix and displaces VEGF-A (particularly the VEGF-A165 isoform), leading to the activation of VEGFR-2 and ultimately forming a positive feedback loop that promotes angiogenesis, inflammation, and vascular permeability (Roudnicky et al., 2013; Rocha et al., 2014).

### 4.5 NF-κB signaling pathway

The NF-κB signaling pathway regulates biological processes such as cell proliferation, apoptosis, metastasis, invasion, and chemoresistance (Wu et al., 2023). It is reported that ESM1 induces AKT phosphorylation to activate IκB kinase (IKK), thereby triggering the phosphorylation and ubiquitin-mediated degradation of IκB proteins. Subsequently, NF-κB dimers translocate to the nucleus to promote cyclin D1 expression, accelerating cell cycle progression and ultimately stimulating cancer cell proliferation (Kang et al., 2012; Liu et al., 2021). Furthermore, PKC may activate the IKK/IκB/NF-κB cascade to promote ESM1 expression in HUVEC (Abid et al., 2006). However, whether a positive feedback loop exists between ESM1 and NF-κB signaling remains to be further explored.

### 4.6 ZEB1/PI3K/AKT signaling pathway

The ZEB1/PI3K/AKT signaling pathway is involved in multiple cancer processes, such as EMT (Qi et al., 2024; Gonzalez-Sanchez et al., 2025). Evidence suggests that ESM1 promotes ZEB1 expression, thereby inducing the phosphorylation of PI3K. Subsequently, activated PI3K phosphorylates AKT, leading to EMT (Qi et al., 2024).

### 4.7 DLL4/Notch signaling pathway

The DLL4/Notch signaling pathway plays a crucial role in cancer cell proliferation (Nakano et al., 2022). Evidence suggests that ESM1 promotes DLL4 expression to activate the Notch pathway. Subsequently, the activated Notch pathway stimulates CDK1 and p21 to accelerate the G2/M-phase cell cycle transition, thereby inducing the proliferation of adrenocortical carcinoma cells (Huang et al., 2021).

### 4.8 PI3K/AKT/FOXO3a signaling pathway

The PI3K/AKT/FOXO3a signaling pathway is involved in regulating cell survival (Sun et al., 2023). It is reported that PI3K activates AKT, leading to the phosphorylation and inactivation of FOXO3a, thereby inhibiting ESM1 expression in HUVEC (Abid et al., 2006).

### 4.9 AKT/eNOS and NF-κB/iNOS signaling pathways

The AKT/eNOS and NF-κB/iNOS signaling pathways play important roles in regulating endothelial function and vascular integrity (Alghanem et al., 2021; Liu et al., 2023). ESM1 inhibits the AKT/eNOS pathway and activates the NF-κB/iNOS pathway to promote oxidative stress in endothelial cells. On the one hand, ESM1 inhibits AKT phosphorylation to block the activation of eNOS, reducing the bioavailability of eNOS-mediated NO. On the other hand, ESM1 activates the NF-κB pathway to stimulate iNOS expression, thereby producing excessive NO and ultimately exacerbating oxidative stress (Kumar and Mani, 2021).

### 4.10 Wnt/β-catenin signaling pathway

The Wnt/β-catenin signaling pathway participates in cellular homeostasis and tumorigenesis (Wang et al., 2024). On the one hand, ESM1 promotes SCD1 expression to activate the Wnt/β-catenin pathway, thereby inhibiting C-C motif chemokine ligand 4 (CCL4) expression, ultimately suppressing the activation of CD8^+^ T-cells to promote immune evasion and cisplatin resistance in ovarian cancer (Fan et al., 2024). On the other hand, nuclear ESM1 binds to the ARM domain of β-catenin to promote the interaction between β-catenin and T-cell factor 4 (TCF4), thereby activating Wnt target genes, ultimately enhancing the stemness and drug resistance of prostate cancer (Pan et al., 2021).

### 4.11 mTOR signaling pathway

The mTOR signaling pathway regulates various biological processes such as cell growth, proliferation, and metabolism (Krencz et al., 2020). ESM1 induces AKT phosphorylation to activate mTOR signaling. Subsequently, mTOR signaling leads to the phosphorylation of ribosomal protein S6 (RPS6) to stimulate the proliferation of VSMCs (Xie et al., 2019). In addition, the ESM1/AKT/mTOR pathway may promote SCD1 and FASN expression, thereby driving lipid metabolic reprogramming in cancer cells (Feng et al., 2024). Moreover, ESM1 may promote PI3K phosphorylation to activate the AKT/mTOR pathway, thereby inducing cell proliferation and angiogenesis in cancer (Yang et al., 2023b).

### 4.12 IRF5/pSTAT1::pSTAT2 signaling pathway

The IRF5/pSTAT1::pSTAT2 signaling pathway plays an important role in inflammation (Shao et al., 2025; Petrova et al., 2026). It is reported that the activation of IRF5 within aortic endothelial cells may promote the phosphorylation of STAT1 and STAT2. Subsequently, phosphorylated STAT1 and phosphorylated STAT2 form pSTAT1::pSTAT2 heterodimers. Then, the heterodimers translocate into the nucleus, thereby binding to the promoter region of the ESM1 gene, increasing ESM1 expression to exacerbate arterial inflammation, cellular apoptosis, and vascular remodeling (Behnoush et al., 2023; Shao et al., 2025).

### 4.13 HIF-1α/VEGF signaling pathway

The HIF-1α/VEGF signaling pathway plays a pivotal role in mediating the cellular response to hypoxia (Zhi et al., 2018; Xu et al., 2023). Under intermittent hypoxia (IH), HIF-1α unmodified by prolyl hydroxylases translocates to the nucleus, thereby binding to HIF-1β to form dimers to recruit p300/CBP. Subsequently, the dimers with p300/CBP bind to hypoxia response elements (HREs), thereby promoting VEGF expression, ultimately enhancing ESM1 level to promote the adhesion of monocytes to endothelial cells (Kim and Lee, 2017; Sun et al., 2019).

## 5 ESM1 and respiratory diseases

Increasing studies suggest that ESM1 is involved in several respiratory diseases, such as lung cancer, chronic obstructive pulmonary disease (COPD), OSAHS, pulmonary arterial hypertension (PAH), and so on (**Figure 3**).

**Figure 3:**
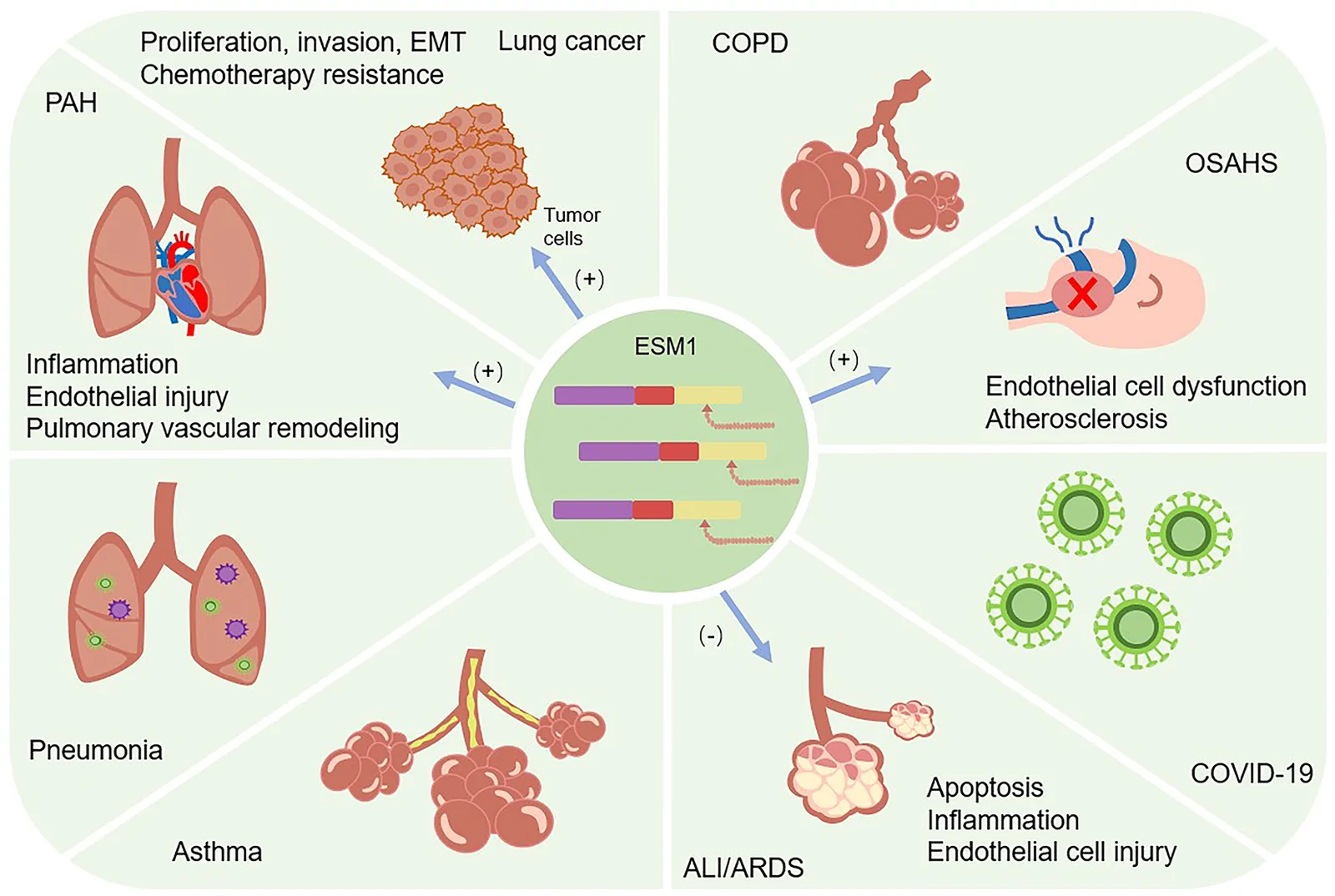
The relationship between ESM1 and respiratory diseases. EMT, epithelial-mesenchymal transition. COPD, chronic obstructive pulmonary disease. OSAHS, obstructive sleep apnea hypoventilation syndrome. COVID-19, coronavirus disease 2019. ALI, acute lung injury. ARDS, acute respiratory distress syndrome. PAH, pulmonary arterial hypertension.

### 5.1 Lung cancer

Lung cancer is the leading cause of cancer-related mortality worldwide (Siegel et al., 2023; Xiao et al., 2023). Emerging evidence indicates that ESM1 plays an important role in lung cancer (Grigoriu et al., 2006; Yang et al., 2015).

It is reported that ESM1 promotes the proliferation, invasion, EMT, and chemoresistance of lung cancer cells (Gu et al., 2021; Aliquò et al., 2023; Kang et al., 2023). Additionally, other authors confirmed that ESM1 upregulates the expression of pro-angiogenic factors within the tumor microenvironment (TME) to promote endothelial cell proliferation, migration, lipid synthesis, and tube formation, ultimately driving angiogenesis (Feng et al., 2024). Accordingly, ESM1 not only enhances the malignant metastasis of cancer cells but also reshapes the TME, thereby promoting cancer progression through autocrine and paracrine mechanisms (Aliquò et al., 2023).

ESM1 promotes lung cancer via several mechanisms. Evidence suggests that ESM1 may induce the expression of long non-coding RNAs (lncRNAs), including H19 and highly upregulated in liver cancer (HULC), thereby promoting cell proliferation and angiogenesis (Aliquò et al., 2023). Meanwhile, the phenylalanine-rich region of ESM1 may interact with immune cell surface receptors (such as LFA-1) to regulate the TME and immune evasion (Scherpereel et al., 2003). Moreover, ESM1 may activate the AKT/mTOR pathway to promote lipid metabolic reprogramming, leading to the proliferation and migration of lung adenocarcinoma cells (Feng et al., 2024). Furthermore, ESM1 may activate the HIF-1α signaling pathway to enhance the stemness and drug resistance of lung cancer stem cells, thereby promoting the proliferation of cancer cells (Gu et al., 2021).

NSCLC is the most prevalent histological subtype of lung cancer (Salloum and Braithwaite, 2022). Studies have reported that the DS chain of ESM1 binds to HGF to activate the ESM1/HGF/c-MET positive feedback loop, thereby promoting cell proliferation and angiogenesis in NSCLC (Béchard et al., 2001a; Li et al., 2019; Yao et al., 2024). In addition, ESM1 activates the EGFR/ESM1 positive feedback loop to promote progression and drug resistance in NSCLC (Yang et al., 2020).

Accordingly, ESM1 may promote lung cancer progression via several signaling pathways, such as the AKT/mTOR pathway, the HIF-1α signaling pathway, the EGFR/ESM1 positive feedback loop, and the ESM1/HGF/c-MET positive feedback loop. However, whether the Wnt/β-catenin, DLL4/Notch, and ZEB1/PI3K/AKT signaling pathways are involved in lung cancer remains unclear. Additionally, the AKT/mTOR and HIF-1α signaling pathways are associated with inflammation, whether ESM1 regulates inflammation in lung cancer via these pathways needs to be further explored. Moreover, how ESM1 regulates H19 and HULC is unclear.

### 5.2 COPD

COPD is a preventable and treatable disease characterized primarily by inflammation, airway remodeling, and emphysema (Uwagboe et al., 2022). It is reported that ESM1 is a target gene of hsa-miR-3685. In COPD, the down-regulation of hsa-miR-3685 relieves the suppression of ESM1, thereby up-regulating ESM1 (Liu et al., 2025).

It is reported that the specific mechanisms of ESM1 in COPD remain controversial (Dai et al., 2018; Zhang et al., 2020; Liu et al., 2025). Some research suggests that ESM1 exerts a protective effect against lung injury (Zhang et al., 2020; Liu et al., 2025). ESM1 may inhibit the apoptosis of pulmonary structural cells and reduce the repair of alveolar cells by enhancing the protective effects of HGF and VEGF on the lung, thereby attenuating emphysema (Zhang et al., 2020). However, other research indicates that ESM1 may facilitate COPD progression. It is reported that ESM1 may induce apoptosis in lung structural cells by up-regulating the Fas/FasL signaling pathway, leading to lung tissue injury and pathological remodeling, ultimately accelerating the decline in pulmonary function (Dai et al., 2018). However, the mechanism underlying the controversial roles of ESM1 in COPD needs to be further explored. Furthermore, whether ESM1 regulates oxidative stress and inflammation in COPD remains unclear.

### 5.3 OSAHS

OSAHS is the most common sleep disorder worldwide (Chen et al., 2022). It is reported that serum ESM1 levels are increased due to the downregulation of miR-181a-1 and decreased after treatment (Altintas et al., 2016; Kanbay et al., 2018; Takasawa et al., 2024). Furthermore, studies suggested that the HIF-1α/VEGF pathway promotes ESM1 expression under IH (Sun et al., 2019). Elevated ESM1 increases vascular permeability, stimulates the expression of ICAM-1 and VCAM-1, enhances leukocyte transendothelial migration, and promotes monocyte-endothelial cell adhesion, thereby exacerbating inflammation (Sun et al., 2019; Altintas et al., 2023; He et al., 2023). Accordingly, ESM1 plays an important role in OSAHS (Sun et al., 2019, 2022; He et al., 2023). However, the specific signaling pathways by which ESM1 promotes OSAHS are unclear.

### 5.4 PAH

PAH is characterized by the occlusion and stenosis of pulmonary arterioles, leading to increased right ventricular afterload and ultimately triggering right heart failure (Rajagopal and Yu, 2022; Sherman et al., 2022). It is reported that serum ESM1 is elevated in PAH (Zhao et al., 2020a, 2020b). Elevated ESM1 may induce EndMT to drive vascular remodeling, thereby promoting PAH (Zhao et al., 2020b). Moreover, studies suggest that ESM1 may induce the expression of VCAM-1, ICAM-1, and E-selectin, leading to endothelial injury and consequently exacerbating PAH (Zhao et al., 2017, 2020a). In addition, recent studies suggest that ESM1 is involved in PAH via a positive feedback loop with TNF-α. ESM1 may stimulate TNF-α expression, further promoting endothelial injury in PAH. Meanwhile, TNF-α may promote ESM1 expression to increase endothelial permeability and stimulate adhesion molecule expression, thereby exacerbating inflammation and pulmonary vascular remodeling in PAH (Zhao et al., 2017). Accordingly, ESM1 may promote the progression of PAH. However, current research is mainly based on animal models and cell experiments. Further exploration is needed to explore the role of ESM1 in patients with PAH. Additionally, the specific mechanisms by which ESM1 drives PAH remain unclear.

### 5.5 ALI/ARDS

ARDS represents a more severe form of ALI (Xie et al., 2025).

Some studies indicate that ESM1 reduces neutrophil infiltration, inhibits LFA/ICAM-mediated leukocyte migration, and decreases inflammatory cytokine secretion, thereby inhibiting inflammation to attenuate ALI/ARDS (Zhang et al., 2018; Gaudet et al., 2019). In addition, ESM1 may restore mitochondrial respiratory function, maintain cellular metabolic homeostasis, reduce apoptosis, and inhibit inflammation by reversing lipopolysaccharide (LPS)-induced overactivation of the mitochondrial unfolded protein response (UPRmt) and metabolic reprogramming, thereby attenuating ALI/ARDS (Zhang et al., 2018). Moreover, ESM1 promotes the repair and regeneration of the endothelial glycocalyx, thereby protecting endothelial cells, maintaining the integrity of the pulmonary vascular barrier, and attenuating ALI/ARDS (Suzuki et al., 2020). Accordingly, ESM1 plays a protective role in ALI/ARDS (Orbegozo et al., 2017; Gaudet et al., 2018; Hureau et al., 2023). However, the specific signaling pathways through which ESM1 exerts protective effects in ALI/ARDS need to be further explored. In addition, ESM1 plays dual pro-inflammatory and anti-inflammatory roles. Whether ESM1 promotes inflammation in ALI/ARDS remains unclear.

### 5.6 COVID-19

COVID-19 is an infectious disease caused by severe acute respiratory syndrome coronavirus 2 (SARS-CoV-2) infection, characterized by high morbidity and mortality (Yuen et al., 2021).

It is reported that ESM1 is elevated in COVID-19 and a higher ESM1 level is associated with increased disease severity of COVID-19 (Görgün et al., 2021; Chenevier-Gobeaux et al., 2022; Keskinidou et al., 2023). In addition, studies suggest that ESM1 may promote the activity of leukocytes and platelets, as well as upregulate the expression of inflammatory cytokines and adhesion molecules to enhance leukocyte adhesion and induce platelet recruitment, thereby promoting thrombosis in COVID-19 (Chenevier-Gobeaux et al., 2022). Accordingly, ESM1 may be related to COVID-19 (Chenevier-Gobeaux et al., 2022; Lipińska-Gediga et al., 2023). However, the relationship between ESM1 and COVID-19 remains unclear and requires further research.

### 5.7 Other respiratory diseases

Pneumonia is an acute infectious disease of the lung parenchyma (Lanks et al., 2019). Evidence suggests that ESM1 exerts anti-inflammatory effects by inhibiting leukocyte recruitment in pneumonia (Perrotti et al., 2018). In addition, ESM1 is helpful in differentiating bacterial from viral pneumonia, and may effectively predict the onset of pneumonia and assess disease severity (Kao et al., 2014; Kupeli et al., 2018; Perrotti et al., 2018; Taşar et al., 2022). Moreover, ESM1 may predict the risk of severe disease in *Mycoplasma pneumoniae* pneumonia (Yu et al., 2025). Accordingly, ESM1 plays an important role in pneumonia. However, the specific roles remain unclear.

Sarcoidosis is a chronic inflammatory granulomatous disease of unknown etiology (Namsrai et al., 2024). Studies indicate that ESM1 not only reflects inflammatory status, but also helps predict vascular involvement and subclinical atherosclerosis in sarcoidosis (Aciksari et al., 2018). However, whether ESM1 is involved in the pathogenesis of sarcoidosis needs to be further explored.

Asthma is a chronic heterogeneous disease of the airways characterized by chronic inflammation and airway hyperresponsiveness (Cevhertas et al., 2020; Dai et al., 2025). Evidence suggests that ESM1 is involved in asthma by regulating microvascular inflammation and endothelial dysfunction (Abdel-Mohsen and Allam, 2018; Tsilogianni et al., 2020). However, the specific roles of ESM1 in asthma require further exploration.

## 6 Conclusion and future perspectives

ESM1 is a soluble dermatan sulfate proteoglycan encoded by the human chromosome 5q11.2 region. ESM1 may regulate inflammation, promote cell proliferation, inhibit programmed cell death, and promote metabolic reprogramming. In addition, ESM1 is involved in several signaling pathways, such as the EGFR/ESM1, EGFR/HER3-Akt/ANGPT2, HGF/c-MET, and VEGF-A/ESM1 signaling pathways. However, the molecular mechanism by which ESM1 regulates inflammation is unclear. In addition, whether ESM1 plays a role in non-neoplastic respiratory diseases by regulating metabolic reprogramming requires more exploration. Moreover, the specific signaling pathways mediating the interaction between ESM1 and TNF-α need to be further explored.

Accumulating evidence suggests that ESM1 plays important roles in respiratory diseases. In lung cancer, ESM1 promotes cancer cell proliferation through the EGFR, NF-κB, and HGF/c-MET signaling pathways. In OSAHS, ESM1 enhances inflammatory factor secretion and monocyte-endothelial cell adhesion through the HIF-1α/VEGF signaling pathway. In PAH, ESM1 may promote vascular remodeling and inflammation via a positive feedback loop with TNF-α. In ALI/ARDS, ESM1 may inhibit leukocyte recruitment to reduce inflammation, while also promoting endothelial injury. However, the molecular mechanism by which ESM1 regulates EndMT in PAH remains unclear. In addition, the specific roles and mechanisms of ESM1 in COPD, asthma, and pneumonia need to be further explored.

Accordingly, ESM1 may serve as a potential therapeutic target for respiratory diseases.
